# Network care: relationship between prenatal care adequacy and hospital obstetric care in a cross-sectional study^*^

**DOI:** 10.1590/1980-220X-REEUSP-2022-0011en

**Published:** 2022-07-01

**Authors:** Fernanda Marçal Ferreira, Kelly Cristina Máxima Pereira Venâncio, Nádia Zanon Narchi

**Affiliations:** 1 Universidade de São Paulo, Escola de Enfermagem, São Paulo, SP, Brazil.; 2 Universidade de São Paulo, Escola de Artes, Ciências e Humanidades, São Paulo, SP, Brazil.

**Keywords:** Health Services, Prenatal Care, Maternal-Child Health Services, Nursing, Servicios de Salud, Atención Prenatal, Servicios de Salud Materno-Infantil, Enfermería, Serviços de Saúde, Cuidado Pré-Natal, Serviços de Saúde Materno-Infantil, Enfermagem

## Abstract

**Objective:**

To assess the relationship between prenatal care adequacy and the demand for hospital obstetric care.

**Method:**

A cross-sectional, quantitative study, conducted in a Brazilian capital, at six basic units and a hospital unit, from 2017 to 2020. Pregnant women who met the predefined inclusion and exclusion criteria participated in the study. Data were collected by structured questionnaire, and follow-up of participants was in person, by phone and by application. Descriptive and analytical statistics were performed using a statistical program.

**Results:**

A total of 224 women were investigated. Prenatal care was adequate in 42.4% of cases, and the mean percentage of adequacy was 76.7% of assessed indicators. Of the 1,067 hospital visits, 63.1% were inopportune. The regression model showed that the variable “prenatal care adequacy” was statistically relevant (0.043), with a 2.2 times higher Odds Ratio (OR) of women who had inadequate prenatal follow-up seeking the hospital inanely.

**Conclusion:**

Prenatal care inadequacy was related to the inopportune search for emergency room care, with care overload for this point in the care network.

## RESUMO

**Objetivo:**

Avaliar a relação entre a adequação do pré-natal e a demanda de atendimentos obstétricos hospitalares.

**Método:**

Estudo transversal, quantitativo, conduzido em uma capital brasileira, em seis unidades básicas e uma unidade hospitalar, de 2017 a 2020. Participaram gestantes que atenderam aos critérios de inclusão e exclusão pré-definidos. Os dados foram coletados por questionário estruturado e o seguimento das participantes foi presencial, telefônico e por aplicativo. Procedeu-se à estatística descritiva e analítica, utilizando um programa estatístico.

**Resultados:**

Foram investigadas 224 mulheres. O pré-natal foi adequado em 42,4% dos casos, e o percentual médio de adequação foi de 76,7% dos indicadores avaliados. Dos 1.067 atendimentos hospitalares, 63,1% foram inoportunos. O modelo de regressão mostrou que a variável “adequação do pré-natal” foi estatisticamente relevante (*p* 0,043), com razão de chance (OR) 2,2 vezes maior de as mulheres que tiveram um acompanhamento pré-natal inadequado buscarem o hospital de forma inoportuna.

**Conclusão:**

A inadequação do pré-natal teve relação com a procura inoportuna por atendimentos em pronto-socorro, com sobrecarga assistencial para este ponto na rede de cuidado.

## RESUMEN

**Objetivo:**

Evaluar la relación entre la adecuación de la atención prenatal y la demanda de atención obstétrica hospitalaria.

**Método:**

Estudio transversal, cuantitativo, realizado en una capital brasileña, en seis unidades básicas y una unidad hospitalaria, de 2017 a 2020. Participaron gestantes que cumplieron con los criterios de inclusión y exclusión predefinidos. Los datos fueron recolectados a través de un cuestionario estructurado y el seguimiento de los participantes fue presencial, telefónico y por aplicación. La estadística descriptiva y analítica se realizó mediante un programa estadístico.

**Resultados:**

224 mujeres fueron investigadas. La atención prenatal fue adecuada en el 42,4% de los casos, y el porcentaje medio de adecuación fue del 76,7% de los indicadores evaluados. De las 1.067 visitas hospitalarias, el 63,1% fueron inoportunas. El modelo de regresión mostró que la variable “adecuación de la atención prenatal” fue estadísticamente relevante (p 0,043), con una razón de probabilidad (OR) 2,2 veces mayor para las mujeres que tuvieron atención prenatal inadecuada para buscar el hospital de manera inoportuna.

**Conclusión:**

La inadecuación de la atención prenatal se relacionó con la demanda inoportuna de visitas a urgencias, con sobrecarga asistencial para ese punto de la red asistencial.

## INTRODUCTION

The organization of care to establish a health care network aims to provide better access, coverage and quality of services offered to the population. With regard to maternal and child health, Brazilian public policy, which aims to accompany from prenatal care, offering from childbirth care to child care, called *Rede Cegonha* (RC), completed more than 10 years of its institution, through ministerial decree published in 2011, with the objective of implementing a new model of care based on reception and resolution with a view to reducing maternal and child morbidity and mortality^(1)^.

National statistics reveal increased coverage in prenatal care, but also significant incidence figures of treatable and/or avoidable morbidities, a trend that has been observed for some years in the country and which is quite contradictory^(2)^. In fact, follow-up adequacy is related to the improvement of maternal and neonatal outcomes, both because of their role in injury prevention during gestational follow-up and the opportune referral to appropriate care services for obstetric complications, reflecting a great deal on the strengthening of health networks and reducing the overload of referral services^(3,4)^.

Elucidating the problem, recent Brazilian statistics indicate significant access to prenatal care with coverage of approximately 98.5% of women, with 72.4% having had more than seven consultations. In the state of São Paulo and in the city of São Paulo, the coverage of this follow-up with more than seven consultations is greater than 80%^(2)^. However, statistics on morbidities highlight the increase in cases of syphilis in historical series from 2010 to 2015, with the trend maintained in the growth of cases of congenital syphilis until 2018 and a minor reduction in 2019^(5)^. This example may apply to other situations where inadequate prenatal care, despite broad coverage and access, leads to unwanted outcomes^(6)^.

In this context, this study aimed to assess the relationship between prenatal care adequacy and hospital obstetric care demand, beginning from the hypothesis that prenatal care inadequacy drives the demand for obstetric emergency services for causes not relevant to this level of care, overloading the units and, consequently, compromising care, in order to generate consequences that can be irreparable to maternal and child health. The answers to the various questions that involve this theme make it possible to expand knowledge about the adequacy of prenatal care offered, as well as its reflection on the dynamics of care in the integrated network of maternal and child health care.

## METHOD

### Design of Study

The article has an observational, analytical, cross-sectional research design with a quantitative approach. The cross-sectional section “produces snapshots of the health situation of a population or community based on individual assessment” has been used in investigative practice, with variations, such as the “study of groups undergoing treatment”, in which comparative groups of exposure and outcome are assessed adopted in this study^(7)^.

### Local

To determine the study area, the premise was assumed that, although the territorial regions and micro-regions of the city of São Paulo have heterogeneous characteristics, there is uniqueness of prenatal guidelines for the municipality. Thus, in the impossibility of studying the entire municipality health care network, in September 2017, the municipality health regions of were mapped by geographic delimitation. The Southeast Regional Health Coordination (RHC) was drawn by simple probabilistic sampling, among the six RHC of the city. The Southeast RHC is subdivided into five Technical Health Supervisions (THS), containing six hospital units, of which four were eligible for having more than 500 childbirths per year (base year 2016), according to data from the Municipal Health Department. Hospital selection also occurred by simple probabilistic sampling, and the Hospital Municipal Vila Santa Catarina was drawn. Then, the 13 Basic Health Units (BHU) referred to this hospital were selected by stratified probability sampling, considering the type of care of each one of them (traditional, Family Health Strategy or mixed), which resulted in the selection of six units: BHU Vila Santa Catarina, with 100% coverage by the Family Health Strategy (FHS); BHU Integrated Americanópolis, with 58.14% FHS coverage; BHU Canaã, with 67.16% FHS coverage; BHU Vila Campestre, with 100% FHS coverage; BHU Integrada Cupecê, with 90.56% FHS coverage; and BHU Jardim Lourdes, with 100% FHS coverage.

### Population and Sample Selection

The study population consisted of pregnant women, regardless of maternal and gestational age, as well as the gestational trimester in which they began prenatal care at the selected BHU, who were willing to participate in the study and agreed to do so. Those who did not follow the pregnancy at the BHU, those who were transferred to units outside the territorial region of study due to change of residential address and those who refused to participate were excluded.

Considering the population of pregnant women in the health units as a dynamic population group, the sample size was calculated using as a basis the results of a pilot study, which had an observational, descriptive, cross-sectional and quantitative approach. The ratio between the participants exposed to adequate and inadequate prenatal care was considered to be 1:1, a significance level (〈) equal to 0.05 and a confidence level of 95%, using the prevalence of events of interest for numerical calculation, which indicated a minimum number of 268 pregnant women for follow-up. The main study involved 273 participants who began prenatal follow-up in the selected health units, and the sampling of participants was not stratified, with loss to follow-up of 17.9%.

### Data Collection

It began in September 2018 and ended in December 2019, when the last participant recruited to compose the follow-up had the pregnancy outcome with the birth of her child. The collection had a dynamic characteristic, so that all women who began prenatal care in selected BHU were approached until the defined sample was obtained. As a follow-up method, aiming to minimize losses, face-to-face contacts were made throughout follow-up, coinciding with the prenatal follow-up routine in services. Also, periodic telephone and telephone contacts were made by application, as agreed with each woman at the time of capture. Finally, regular visits were made to the maternity ward to verify the records of emergency obstetric care of participants.

**Figure 1.**
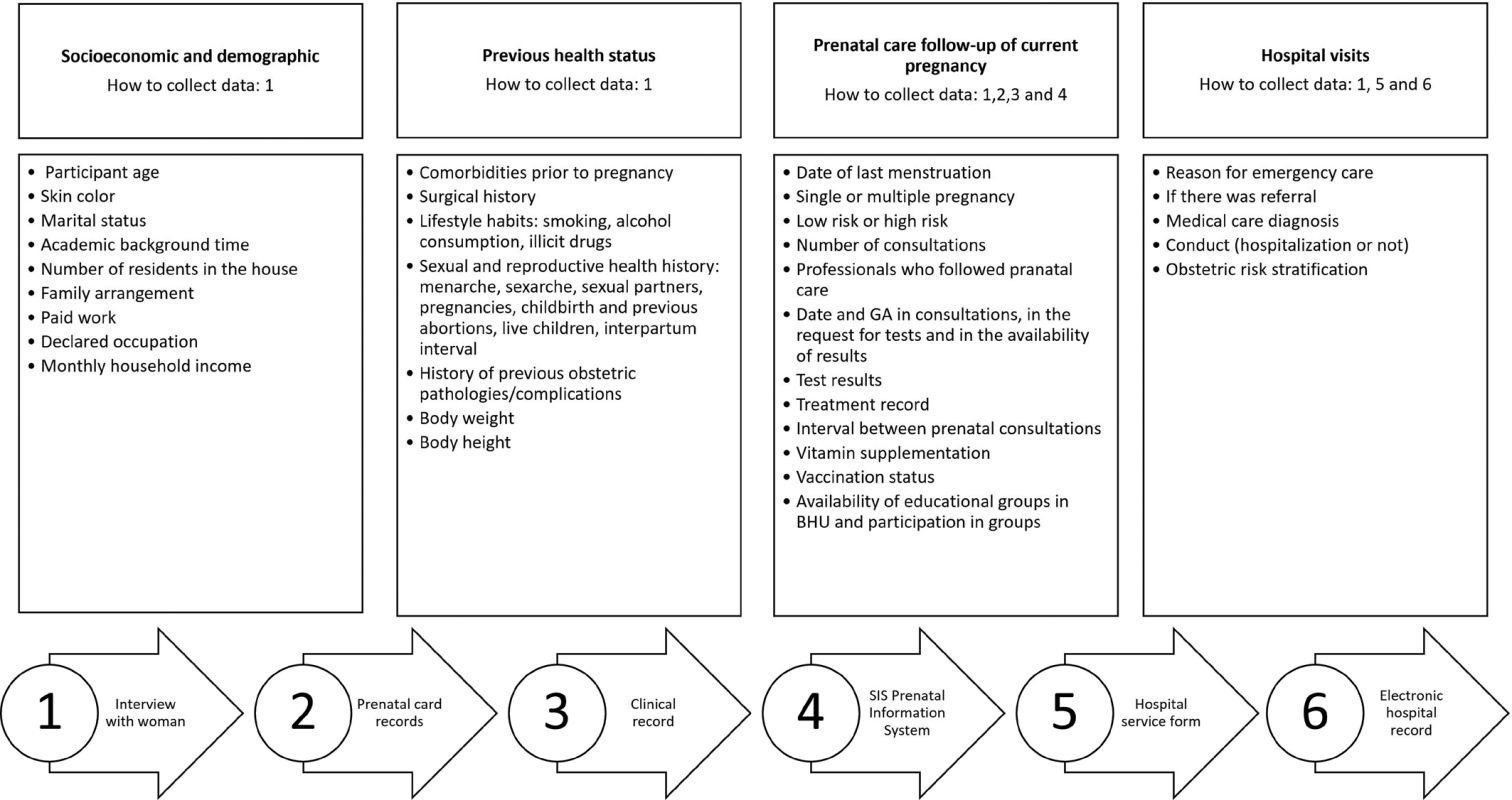
Study variables and information sources.

*Source*: authors.

The data collection instrument was submitted to validation by judges with expertise in maternal and child health, and was previously tested to observe its applicability, relevance, understanding and internal consistency. It was constituted in a structured form, whose variables and respective forms of collection are presented in Figure 1.

**Figure 2.**
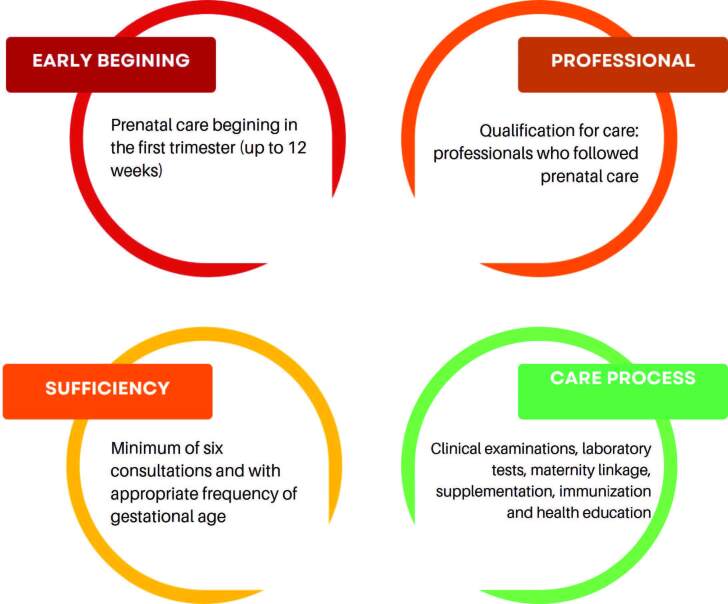
Criteria for assessing prenatal care according to the dimensions proposed by Heredia-Pi et al^(12)^.

*Source*: authors.

The criteria adopted for prenatal care adequacy assessment (Figure 2) and hospital care classification as “opportune” or “inopportune” were the use of the Primary Care Protocol^(8)^ and the Manual of Reception and Risk Stratification in Obstetrics^(9)^, respectively. It is added that the indicative signs of referral to emergency obstetric care are also part of obstetric care municipal protocol.

Following literature parameters^(10,11)^, values above 80% were adopted as the cut-off point to consider prenatal care adequacy. For the set of hospital visits per woman, the same cut-off point was adopted to maintain coherence between the analyzed processes, since the literature only assesses the pertinence of occasional visits.

### Data Analysis and Treatment

The information was entered into Excel Microsoft^®^ 365 spreadsheets, with double-entry typing for agreement assessment, verification and correction of insertion errors. The database was transported to the IBM^®^ SPSS^®^ Statistics program, version 25. Descriptive statistical analysis was performed using absolute and relative frequency distributions and measures of centrality and variability, unadjusted bivariate and adjusted multivariate analysis, including measures of association in contingency tables (X^2^, RR and OR) and multiple binomial logistic regression. A significance level of alpha (〈) was considered equal to 0.05 (95% confidence interval).

### Ethical Aspects

The study followed the ethical principles of Resolution 466/12 of the Brazilian National Health Council for research involving human beings, with ethical approval by the Research Ethics Committees of the *Universidade de São Paulo* Nursing School (Opinion 2,490,668 of 02/07/2018), the Municipal Health Department of the City Hall of São Paulo (Opinion 2,548,667 of 03/18/2018) and the *Hospital Israelita Albert Einstein*, administrator of the investigated hospital institution (Opinion 2,763,011 of 07/10/2018).

## RESULTS

The 224 participants were on average 25.9 years old. Most of them declared black or brown skin color (73.7%) and were married or lived in a stable union partnership (75.4%). The majority (56.7%) did not have a paid job, taking care of household chores and caring for family members (46.4%) or were students (10.3%). As for education, the mean of years of formal schooling without repetition was 9.9 years, equivalent to complete elementary school education. Of the 147 who had had at least one previous pregnancy, 62 (42.2%) had some previous obstetric complication. Regarding gestational risk, 77.2% (n = 224) were classified by prenatal professionals as low-risk pregnancy. Of the 51 women referred for high-risk prenatal care, the pathological obstetric condition was confirmed by the specialized reference for 80.4%. Those who had the risk discarded were referred back to low-risk prenatal care according to counter-referral in the care network (19.6%).

Prenatal care begun on average at gestational age of 10 weeks and 1 day, and the majority (78.1%) began follow-up in a timely manner until the 12^th^ gestational week (n = 224); 21.0% did so in the second quarter; and 0.9% did only in the third. Thus, 87.9% of women had six or more prenatal visits, and 59.4% had no shortage of consultations throughout pregnancy. The means of consultations and absenteeism are presented in Table 1.

**Table 1.** Prenatal care and absenteeism (N = 224) – São Paulo, SP, Brazil, 2018–2019.

Variable	Mean	Median	Standard deviation	Breadth	Minimum	Maximum
Prenatal care	8.61	9.00	2.695	14	1	15
Absenteeism	1.70	1.00	1.071	6	0	6
1^st^ tri* consultations	1.45	1.00	1.019	6	0	6
2^nd^ tri* consultations	2.86	3.00	1.118	6	0	6
3^nd^ tri* consultations	4.57	5.00	1.946	11	0	11

*Gestational trimester.

Prenatal care, considering the three gestational trimesters, was adequate for 42.4% of participants (N = 224). Analyzing follow-up adequacy for the first trimester, 67.4% had adequate prenatal care (N = 224), while for the second and third gestational trimesters, there was adequacy for 35.5% (N = 217) and 46.3% (N = 214), respectively. The overall prenatal care and gestational trimester adequacy is shown in Table 2. It is important to add that the socioeconomic, demographic and clinical-obstetric predictor variables did not show statistical significance for the outcome “prenatal care adequacy or not”. The number of participants (N) in the first trimester of pregnancy reflects the adequacy assessment of those women who effectively underwent follow-up at this time of pregnancy (78% of the total). In the other trimesters, prenatal follow-up discontinuity is reflected by interruption of pregnancy by abortion or premature childbirth.

**Table 2.** Centrality measures for overall prenatal care adequacy and gestational trimester – São Paulo, SP, Brazil, 2018–2019.

Prenatal care adequacy	N	Omitted	Mean	Median	Standard deviation	Breadth
Overall prenatal care	224	0	76.709	77.410	10.3270	73.3
Prenatal care in the 1^st^ tri*	174	50	86.090	87.500	9.1988	73.3
Prenatal care in the 2^nd^ tri*	217	7	73.201	77.770	18.0135	100.0
Prenatal care in the 3^rd^ tri*	214	10	73.504	77.950	16.5746	100.0

*Gestational trimester.

Women (N = 224) used the hospital service, totaling 1,067 consultations, with a mean of 4.75 consultations per pregnant woman. The motivation to seek care at this level of health care was spontaneous for most women (82.2%), and the third gestational trimester was the moment with the highest demand (64.9%). Among the reasons for consultations, pain in the lower abdomen/colic (44.4%), uterine contraction (12.8%), vaginal bleeding (12.7%) and epigastric abdominal pain in flanks and hypochondria (12%) stand out. It is noteworthy that obstetric emergency care resulted in hospitalization in 224 service visits (21%). According to the criteria defined for the research, 394 (36.9%) hospital visits were classified as opportune (36.9%), and 673 (63.1%) as inopportune. Considering opportune care, the mean was 1.75 visits per pregnant woman, and the mean of inopportune care was 3.0 visits to the hospital service per pregnant woman. It was found that only 35 participants sought hospital service in a timely manner (in at least 80% of their visits to the emergency room), which represents 15.6% of the total number of women followed up.

The multivariate analysis by binomial logistic regression for the outcome “inopportune hospital care” is presented in Table 3. Eight predictor variables were used for the regression, obtaining a regression model with good fit to the data, according to p-value for the significance of the Hosmer and Lemeshow test (p 0.746). The variable “prenatal care adequacy” was statistically relevant in the regression (p 0.043), with an OR 2.2 times higher for women who had inadequate prenatal care to seek hospital services in an inopportune manner.

**Table 3.** Multivariate analysis by binomial logistic regression for the outcome “inopportune hospital care” – São Paulo, SP, Brazil, 2018–2019.

Variable	p (X^2^)	OR	(95%) CI		p (Hosmer and Lemeshow test)
			Lower	Upper	
Having/living with a partner	0.490	1.412	0.530	3.760	0.746
Educational level	0.126	0.519	0.224	1.202	
Skin color	0.433	1.396	0.606	3.215	
Paid work	0.541	1.299	0.561	3.006	
Health condition/disease	0.789	1.128	0.466	2.733	
Care modality	0.083	2.492	0.886	7.004	
High-risk pregnancy	0.560	0.760	0.303	1.910	
Prenatal care adequacy	0.043	2.271	1.028	5.020	

## DISCUSSION

The results show that the beginning of prenatal care was early, which denotes the system effectiveness in attracting women to enter the health network, as well as meeting one of the recommendations that make up the prenatal quality standard, which is the access of pregnant women ideally in the first gestational trimester^(13)^.

Quantitative studies that analyzed the variable “early beginning of prenatal care” found results with percentages lower than those of this study, with most of these values varying around 50% and a negative highlight for a case in which the percentage was only 26.3%^(14,15,16)^. It is inferred that the divergence between literature results and those found here may be related to the wide coverage by FHS, as well as by the municipal care protocols. However, it is clear to say that there are great differences between the assistance realities in the other regions of the city, depending on the characteristics of its population and, even, on the outsourced managers of primary care services (PC) in the municipality.

In São Paulo, unlike in other contexts in the country, the articulation between CP and specialized care uses referral criteria defined by protocol, and the consultation scheduling mechanism takes place through two computerized systems, one municipal and the other state. These local network particularities may have been decisive for the good performance in the dimensions of access and link analyzed in a comparative study between Brazilian capitals^(17)^. Corroborating the quantitative findings of this study, São Paulo demonstrates the network’s capacity for responsible referrals, and the role of PC deserves to be highlighted in this articulation, since adequate screening and the time between referral and specialized care can be decisive for the potential risk of pregnant women’s health condition^(18)^. Additionally, maintaining the linkage of pregnant women in PC as the coordinator of care through the care network is essential to qualify care for high-risk pregnant women^(17)^. In contrast to the positive quantitative results found for the network articulation in situations in which women’s health presents complications due to pre-existing conditions or pregnancy complications, there were occasional records of weaknesses in this line of care, especially with regard to the follow-up of pregnant women who were linked to high-risk prenatal care and who required care in more than one specialized service.

Along with the criterion referring to the opportune beginning of prenatal care, the number of consultations during pregnancy follow-up is one of the main indicators for assessing access to and coverage of prenatal care. The comparison of studies on this issue can be hampered, because some studies do not consider the adjustment for gestational age or the interval between consultations according to gestational age. In any case, it is possible to have an overview of care reality, although it is relevant to point out that the number of consultations does not necessarily guarantee its quality.

Other studies found values close to the findings in this study, with percentages ranging from 86.2% to 89%^(19,20)^. The result of this study is expressive, especially if we consider the mean absenteeism of 1.7 consultations per woman, which reveals effectiveness in follow-up and bonding of pregnant women despite the absences, although it also raises the discussion about the reasons attributed to such absences and what measures could be adopted to ensure optimization of care in the care network.

Just as the comparison between published studies on access to prenatal care has limitations related to the different data collection methodologies adopted in these studies, for procedures performed during prenatal care and for comparisons of prenatal care adequacy, this difficulty exists. Nevertheless, statistics and the results of other research can contribute to the discussion of care reality and to proposals for qualification of the maternal and child health care network.

Statistics from the SUS Department of Informatics (DATASUS – *Departamento de Informática do SUS*) show prenatal care “adequacy” and “more than adequacy” for 81.2% of visits to women who gave birth to live newborns in the city of São Paulo in 2018 and 2019^(21)^. The expressive difference found between the municipality’s official statistics and the study’s sample point to the need to discuss the criteria adopted to assess service quality.

Data point to a care reality with good coverage of access to prenatal care, and the Ministry of Health index is, in fact, relevant to assess the adequacy of access to care during pregnancy. Perhaps this is a sign that we must advance in the qualification of indicators, proposing questions such as: would it be possible that the number of consultations and the opportune recruitment of women to begin prenatal care actually reflect the quality of care provided? What is the relevance of this information alone for the implementation of improvements in care and the health care network? And how would it be possible to improve the parameters?

If we compare data susus statistics to another study, which assessed the quality of prenatal care based on data from the main and most recent national survey available **–** considering in its analysis the beginning of prenatal care, the number of consultations, the reference maternity recommendation and the performance of at least one recommended examination per trimester – we will also find large discrepancies, as the minimum overall adequacy was 21.6% for the country and 24.9% for the Southeast region^(22)^. However, if we only assess the opportune beginning of prenatal care or the number of consultations, this difference decreases considerably, with percentages of 53.8% and 86.5%, respectively, for the national base and of 58.2% and 80.5 %, respectively, for the Southeast^(22)^. This same trend was observed in the present investigation.

A cohort study carried out in northeastern Brazil about prenatal care content inadequacy used a hierarchical model for analysis that considers characteristics that are close to what was proposed in this research, having as adequacy variables the beginning of prenatal care, the number of consultations, vaccination, laboratory tests and obstetric procedures, whose results showed that the recommended criteria were not incorporated into clinical practice^(23)^. Other investigations on the prenatal care process indicate a prevalence of around 24% for overall adequacy, including as procedures, exams and orientations received during prenatal care in the basic health network as variables^(19,20)^. The findings of such studies corroborate the results presented here, which show even higher prevalence of inadequacy in the second and third trimesters of pregnancy, when compared to the first trimester of pregnancy.

The relationship between prenatal care and hospital care adequacy in the binomial logistic regression confirms the research hypothesis that inadequate follow-up during pregnancy leads women to seek hospital services for reasons that are inopportune for that level of care. Since other statistically significant correlations have not been demonstrated, it is suggested that studies on the subject continue to be developed, inferring that the limitation in sample size may have been reflected in these results. The articulation and integration of RAS, with qualification of care at all points of care, aiming at reception and prenatal care, are challenges that persist for the improvement of care for pregnant women, which has a direct impact on maternal and child morbidity and mortality outcomes^(24)^.

The present study had as limitations the non-stratified sampling of study participants, the loss of follow-up of women in the monitored group and the diversity of process and outcome indicators for prenatal care assessment found in literature. Furthermore, there is a scarcity of research that investigates the care network, from the perspective of people’s dynamics in health services, in a thesis, articulated. Further investigations can be proposed to understand aspects, such as individual decision-making for the search for emergency obstetric care services, diagnoses on the health network and intervention plans to improve articulation, which could facilitate the movement of people through the network and its effectiveness, in order to reduce the overload of services for causes not relevant to that level of care.

The present study provides an overview that can contribute to the role of nursing in women’s health in the pregnancy-puerperal cycle, since understanding the weaknesses of the health care network can foster strategies to improve process indicators, as well as the quality of prenatal care, especially in health education and health promotion activities, performed mostly by PC nurses.

## CONCLUSION

Prenatal care, which should comply with all procedures and conduct recommended by care guidelines, had percentages pointing to a decline in adequacy in the second and third trimesters of pregnancy in relation to the beginning of follow-up. The overall adequacy found revealed weaknesses of care, although the parameter adopted as a cut-off point was high in relation to literature. Prenatal care indicator assessment allows us to infer about important weaknesses in the development of practices that should be recommended in the monitoring of pregnant women.

Most hospital visits were inappropriate for that level of care, considering hospital admission as an outcome, which leads to concerns for future research related to health education and trust in the level of care as a strategy for individual decision-making about the search for a service.

The results made it possible to assess the maternal and child care network and to conclude that, for the investigated group, prenatal care inadequacy was related to the inopportune demand for consultations in the obstetric emergency room, with damage to the maternal and child health care network, since this overloads a point of care in the care network with a demand that could be solved at another level of care.

Carrying out a situational diagnosis of the care network, at a level of proximity to the local reality, has already contributed to the redefinition of management strategies in some of the health units of this research, which was an important practical implication and contributed to a positive impact on services.

There is a scarcity of research that addresses the care network investigation from the perspective of the dynamics of people in health services, which should, in theory, be articulated. Further studies can be proposed to understand aspects, such as individual decision-making for the search for emergency obstetric services, in addition to others that appropriate diagnoses about the health network and propose intervention plans to improve its articulation, facilitating the movement of people through the network and reducing the overload of services for causes not relevant to that level of care.

## ASSOCIATE EDITOR

Maria Luiza Gonzalez Riesco
